# Gauging the skin resident Leishmania parasites through a loop mediated isothermal amplification (LAMP) assay in post-kala-azar dermal leishmaniasis

**DOI:** 10.1038/s41598-022-21497-6

**Published:** 2022-10-27

**Authors:** Prakash Ghosh, Rajashree Chowdhury, Shomik Maruf, Albert Picado, Faria Hossain, Sophie I. Owen, Rupen Nath, James Baker, Md Golam Hasnain, Mohammad Sohel Shomik, Debashis Ghosh, Masud Rashid, Md. Utba Rashid, Soumik Kha Sagar, Md. Abu Rahat, Ariful Basher, Proggananda Nath, Thomas Edwards, Jason R. Andrews, Malcolm S. Duthie, Dziedzom K. de Souza, Emily R. Adams, Joseph Ndungu, Israel Cruz, Dinesh Mondal

**Affiliations:** 1 Nutrition and Clinical Services Division, icddr,b, Dhaka, 1212 Bangladesh; 2 Foundation for Innovative New Diagnostics (FIND), Geneva, Switzerland; 3 Department of Tropical Disease Biology, Liverpool School of Tropical Medicine (LSTM), Liverpool, UK; 4 School of Medicine and Public Health, The University of Newcastle, Callaghan, NSW Australia; 5 National Heart Foundation and Research Institute, Mirpur, 1216 Bangladesh; 6 Infectious Disease Hospital, Mohakhali, Dhaka, 1212 Bangladesh; 7 Infectious and Tropical Medicine Department, Mymensingh Medical College and Hospital (MMCH), Mymensingh, 2200 Bangladesh; 8 Stanford University School of Medicine, Stanford, CA 94305 USA; 9 HDT Bio, Suite 280, 1616 Eastlake Ave E, Seattle, WA 98102 USA; 10 Department of Parasitology, College of Health Sciences, Noguchi Memorial Institute for Medical Research, University of Ghana, Legon, Ghana; 11 National School of Public Health, CIBERINFEC, Instituto de Salud Carlos III ES, Madrid, Spain

**Keywords:** Biological techniques, Microbiology

## Abstract

Despite the availability of highly sensitive polymerase chain reaction (PCR)-based methods, the dearth of remotely deployable diagnostic tools circumvents the early and accurate detection of individuals with post-kala-azar dermal leishmaniasis (PKDL). Here, we evaluate a design-locked loop-mediated isothermal amplification (LAMP) assay to diagnose PKDL. A total of 76 snip-skin samples collected from individuals with probable PKDL (clinical presentation and a positive rK39 rapid diagnostic test (RDT)) were assessed by microscopy, qPCR, and LAMP. An equal number of age and sex-matched healthy controls were included to determine the specificity of the LAMP assay. The LAMP assay with a Qiagen DNA extraction (Q-LAMP) showed a promising sensitivity of 72.37% (95% CI: 60.91–82.01%) for identifying the PKDL cases. LAMP assay sensitivity declined when the DNA was extracted using a boil-spin method. Q-qPCR showed 68.42% (56.75–78.61%) sensitivity, comparable to LAMP and with an excellent agreement, whereas the microscopy exhibited a weak sensitivity of 39.47% (28.44–51.35%). When microscopy and/or qPCR were considered the gold standard, Q-LAMP exhibited an elevated sensitivity of 89.7% (95% CI: 78.83–96.11%) for detection of PKDL cases and Bayesian latent class modeling substantiated the excellent sensitivity of the assay. All healthy controls were found to be negative. Notwithstanding the optimum efficiency of the LAMP assay towards the detection of PKDL cases, further optimization of the boil-spin method is warranted to permit remote use of the assay.

## Introduction

Post-kala-azar dermal leishmaniasis (PKDL) is a non-fatal complication that typically manifests in people treated for visceral leishmaniasis (VL), but can also develop in individuals without history of VL^1,2^. PKDL presents as a hypo-pigmented skin rash or lesions, commonly involving the emergence of macules on the face, with further development of papules and nodules in the skin, or as polymorphic lesions which subsequently spread across the skin, mostly on the trunk, legs, and genital organs^3,4^. Although this dermatological complication is not life threatening, PKDL patients face severe cosmetic stigma, especially amongst unmarried women^5^.In Sudan, PKDL develops in 50–60% of VL patients within 6 months following completion of their treatment^6–8^. On the Indian subcontinent (ISC), occurrence is less frequent but 5–10% of cured VL patients develop PKDL within 2–4 years^9^. In addition, 15–20% of PKDL patients present without history of VL, suggesting that these individuals may have had an asymptomatic *Leishmania donovani* infection^6^.

A population-based study in Bangladesh previously reported a two-fold increase in the incidence of PKDL within 5 years of VL treatment^6^. Moreover, a recent study by Mondal et al.^9^ identified that varying VL treatments have a significant impact on the development of PKDL. For patients treated with either sodium stibogluconate (SSG) or multi-dose liposomal amphotericin B, incidence of PKDL within 4 years was 3% and 8.2%, respectively^9^. People with PKDL are considered to be a major contributor of inter-epidemic transmission of *L. donovani* and the propagation of new VL cases. A recent study in Bangladesh found that the lesional-skin of PKDL patients harboring *Leishmania* parasites is approximately 58% infectious towards the sand-fly vector, highlighting the need for early and effective PKDL diagnosis and treatment^10^.

Diagnosis of PKDL is challenging, however, with skin lesions and rashes often confused with alternative dermatologic conditions, including vitiligo, leprosy, secondary syphilis, and sarcoidosis^11,12^. There remains no gold-standard technique for diagnosing PKDL, where the diagnosis largely depending on a clinical history coupled with examination by an experienced clinician^3^. PKDL is then traditionally confirmed by microscopic observation of amastigotes in slit skin or skin biopsy smears. However, the sensitivity of conventional microscopy is unsatisfactory, ranging from 67 to 100%, 36–69%, and 7–33% in nodular, papular, and macular PKDL, respectively^11,13,14^. Several serological assays used in VL diagnosis, such as the direct agglutination test (DAT), rK39 enzyme-linked immunosorbent assay (ELISA), and rK39 rapid diagnostic test (RDT) have been used to overcome the limitations of microscopy-based diagnosis of PKDL. These tests are highly sensitive in detecting anti-leishmanial antibodies, particularly the rK39 RDT, and have been adopted in the diagnostic algorithm as an ancillary test for PKDL. However, serological assays alone are inconclusive for the diagnosis of PKDL, as anti-leishmanial antibodies can remain in the circulation for extended periods of time following VL cure^15,16^.

Since molecular methods detect current infection and can be applied to a wide range of clinical specimens, therefore, a handful of molecular-based diagnostic methods such as conventional polymerase chain reaction (PCR) and quantitative real-time PCR (qPCR) have been developed which yielded with promising sensitivities and specificities among VL and PKDL patients^17–19^. Conventional PCR on skin samples was found to have high sensitivity (94.5%) for diagnosis of PKDL, but lacks parasite quantification^20^. More recently, qPCR has emerged as a highly sensitive molecular diagnostic method for detecting VL and PKDL and allows quantification of parasites. Sensitivity of qPCR was 85% in DNA extracted from archived skin biopsies^19^. Further, we validated a real-time PCR assay by assessing its diagnostic efficacy relative to conventional microscopy, finding that an augmented sensitivity of 91.2% for macular cases could be attained if multiple skin samples are used^14^. However, the need for well-resourced laboratories, expensive reagents, a long execution time, and high contamination rates are currently prohibitive for the use of PCR-based assays in resource-limited settings outside of research.

In an effort to address the limitations of the PCR-based diagnostic tools, a loop-mediated isothermal amplification (LAMP) assay (Eiken Chemical Co. Ltd., Japan) has shown promise for detection of *L. donovani* parasites both in blood and tissue^21–23^. This rapid and simple molecular technique has spurred a demand for the deployment of the point-of-need diagnostics at resource-limited settings for leishmaniasis. To date, several LAMP assays have been developed and validated for diagnosis of PKDL in the ISC^24^ and a recent LAMP assay showed promising efficiency using blood samples todiagnose PKDL^25^. However, there are no data on the diagnostic accuracy of the Loopamp™ *Leishmania* Detection Kit (Eiken Chemical Co. Ltd., Japan) for detecting *L. donovani* DNA in skin samples of PKDL patients. In this study, we report the diagnostic efficacy of the Loopamp™ *Leishmania* Detection Kit for diagnosis of PKDL using DNA extracted from skin-snip samples. We used two different analysis approaches to determine diagnostic accuracy, using the existing diagnostic and clinical algorithms as the reference standard, and we performed a latent class analysis (LCA) to determine the sensitivity and specificity of LAMP. Furthermore, we explored the efficacy of a laboratory-improvised rapid DNA extraction method to facilitate the application of LAMP in resource-limited settings.

## Results

### Study participants

**Figure 1:**
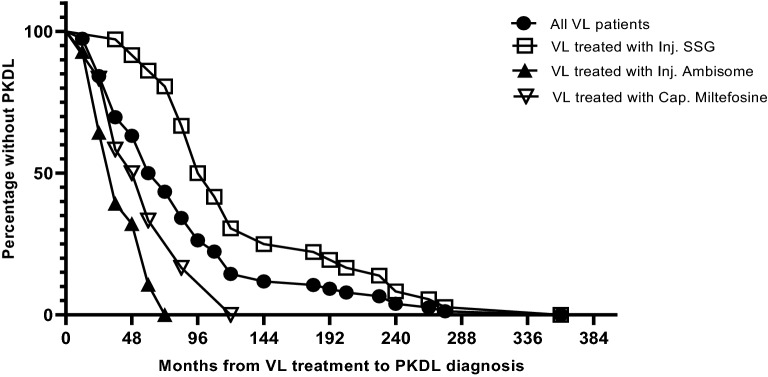
The time (in months) interval from the treatment of visceral leishmaniasis (VL) to being diagnosed as post-kala-azar dermal leishmaniasis (PKDL).

Seventy-six probable PKDL cases were enrolled, all of whom had previously been treated for VL and responded to PKDL treatment: a retrospective diagnosis of PKDL could therefore be reasonably assumed. Having been previously diagnosed with VL, all patients (76/76) had been treated with anti-leishmanial drugs including SSG (47.3%), AmBisome (36.8%), and miltefosine (15.8%). The median period of developing PKDL after successful VL treatment was highest for SSG (8.5 years), followed by miltefosine (4.5 years), and AmBisome (3 years) (Fig. 1). PKDL patients had a mean age of 28.9 (± 15.0 years), and 45 (59.2%) were male. Most presented with macular rash (93.4%), and 5 (6.6%) presented with mixed lesions/rashes (Table 1). Median PKDL rash scores were 35.5 (IQR: 10.3–80.8).

**Table 1:** Demographic and clinical parameters of all of the PKDL cases enrolled in the study.

Variables			
Sex	Male	45 (59.2%) 31 (40.2%)	
	Female		
Age	2 year to < 18 years	20 (26.3%) 11.75 ± 3.26	28.87 ± 14.99
	≥ 18 years	56 (73.7%) 34.98 ± 12.57	
Past history of VL		76/76 (100%)	
Treatment option for VL	Inj. SSG	36/76 (47.37%)	
	Inj. Ambisome	28/76 (36.84%)	
	Cap. Miltefosine	12/76 (15.79%)	
Interval (VL treatment to diagnosis of PKDL) years		5.50 (IQR: 3–9)	
Interval (VL treatment to diagnosis for PKDL) years	Inj. SSG	8.5 (IQR: 7–14.25)	
	Inj. Ambisome	3 (IQR: 2–5)	
	Cap. Miltefosine	4.50 (IQR: 3–7)	
Present PKDL rash scores		35.50 (IQR: 10.25–80.75)	
Type of rashes	Macular(M)	71/76 (93.4%)	
	Nodular(N)	0/76 (0%)	
	Papular(P)	0/76 (0%)	
	Mixed (NM/MPN/MP)	5/76 (6.6%)	

**Figure 2:**
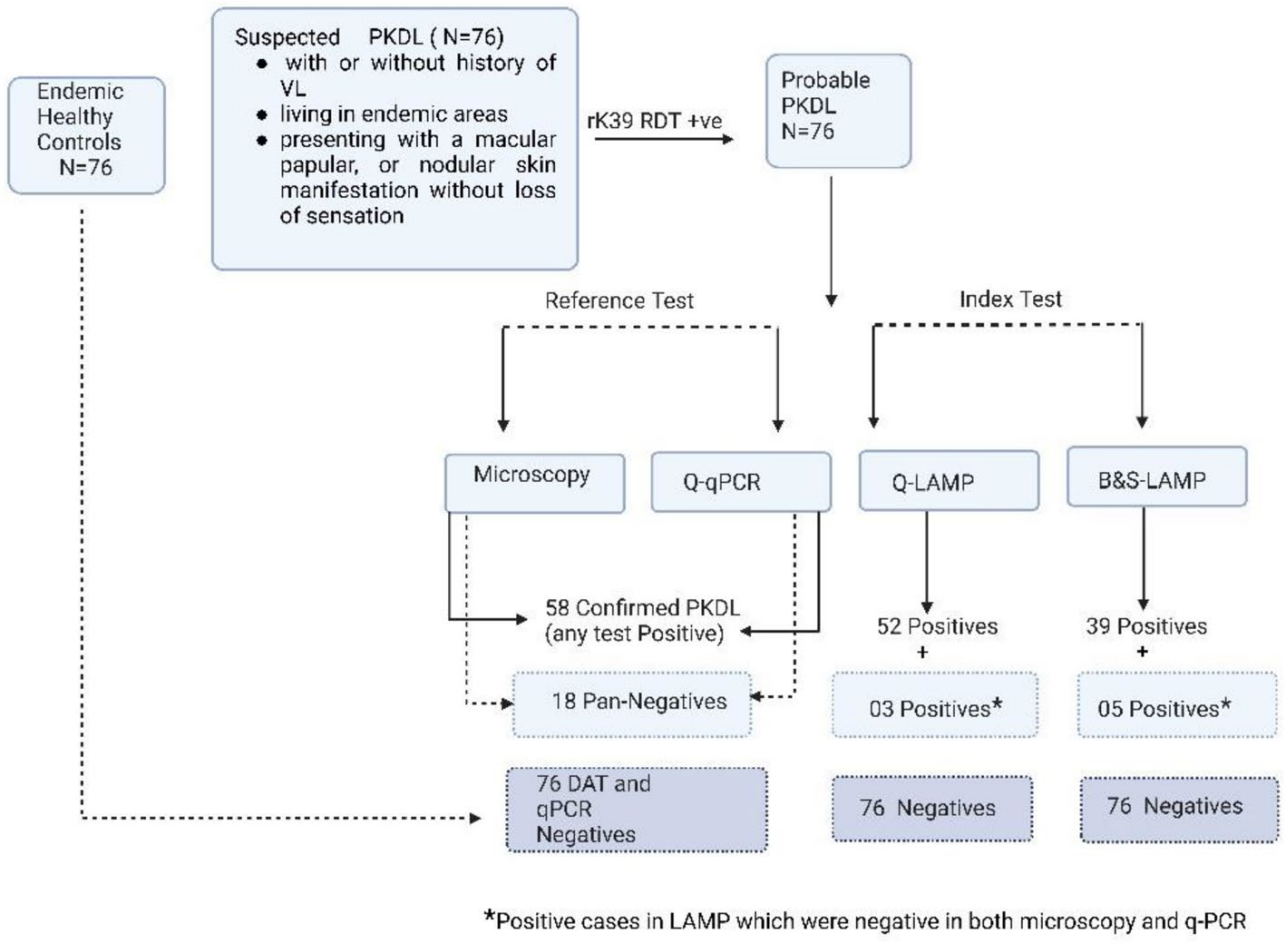
Study flow-diagram representing the inclusion of study participants and eventual diagnosis through index and reference tests.

**Figure 3:**
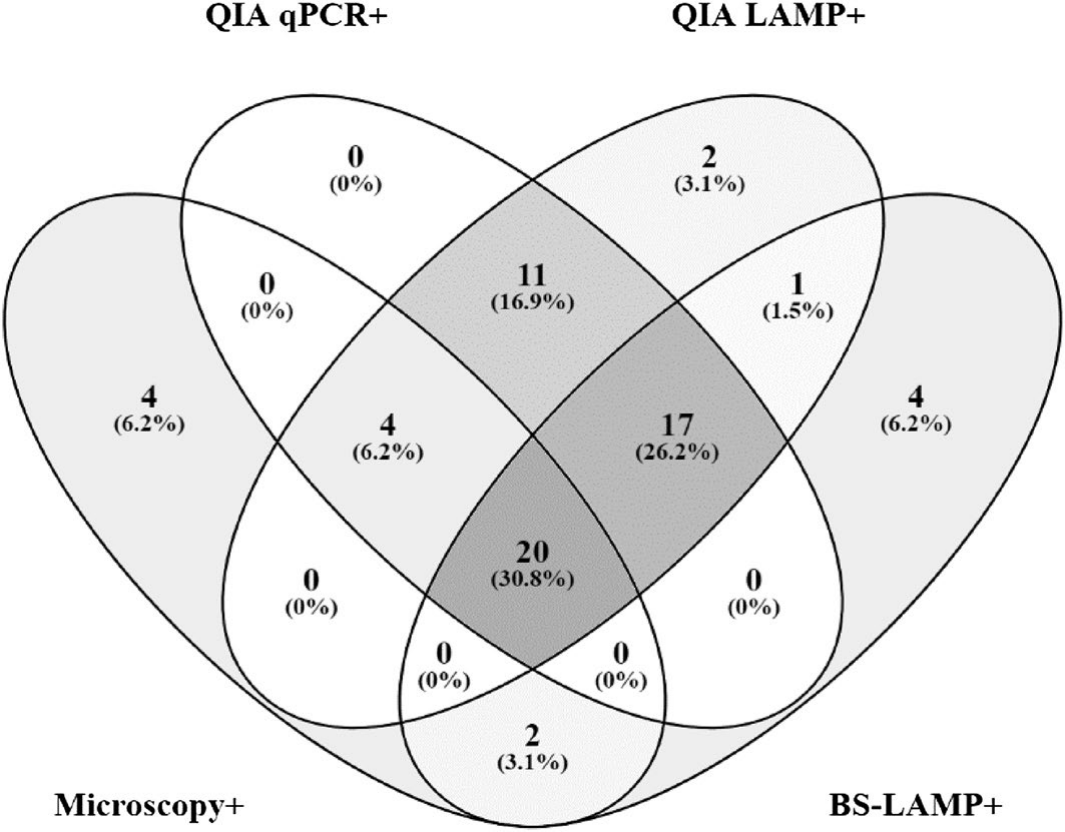
Venn diagram depicting the distribution of 76 probable PKDL cases diagnosed through four different methods. Among 76 probable patients, 20 (30.8%) cases were positive by all of the diagnostic methods where 65 cases were positive by at least one method. Microscopy detected 30 cases as positive where 4 cases were solely detected through Microscopy. Q-qPCR was positive in 52 (68.42%) cases and that 52 cases were positive by at least one other method. Q-LAMP was positive in 55 (72.37%) PKDL patients, 2 (3.1%) of whom were positive for Q-LAMP only. B&S-LAMP was positive in 44 (57.89%) cases, where 4 (6.2%) cases were exclusively positive through this assay only. 38 cases were positive by both Q-LAMP and B&S-LAMP assay where 7 cases were exclusively detected through LAMP assay.

Among the 76 PKDL patients enrolled, 58 (76.3%) cases were confirmed by skin biopsy (qPCR and/or microscopy positive) (Fig. 2). Among these 58 biopsy-confirmed PKDL cases, 28 (48.3%) were qPCR positive, 6 (10.3%) were microscopy positive, and 24 (41.4%) were positive in both assays (Fig. 3). According to microscopy grading system for LD bodies (World Health Organization 1991 splenic aspiration microscopic report grading system), 24 (80.0%) patients were assessed as grade 1, four (13.3%) as grade 2, and one each (3.4%) as grades 4 & 5.

### Diagnostic performance

In participants with confirmed PKDL (n = 58), the sensitivity of LAMP assay performed with DNA extracted using Qiagen kit (Q-LAMP) was 89.7% (95% CI: 78.83–96.11%) compared to 67.0% (95% CI: 53.66–78.99%) for the LAMP assay performed with DNA extracted using the boil & spin technique (B&S-LAMP) (Table 2). Q-LAMP had a specificity of 96.8% (95% CI: 91.0–99.3%) and B&S-LAMP 94.7% (95% CI: 88.0–98.3%) (Table 2). Q-LAMP showed good agreement (k = 0.873) while B&S-LAMP had moderate agreement (k = 0.649) with qPCR assay performed with DNA extracted using the Qiagen kit (Q-qPCR).

**Table 2:** Determining diagnostic efficiency of microscopy, qPCR and LAMP based on the PKDL case definition.

PKDL case definition	Assay type	Sensitivity (95%CI) (n/N)	Specificity (95%CI) (n/N)
Confirmed PKDL (N = 58)	Q-LAMP	89.66% (78.83%—96.11%) (52/58)	96.81% (90.96%-99.34%) (91/94)
	B&S-LAMP	67.24% (53.66%-78.99%) (39/58)	94.68% (88.02%- 98.25%) (89/94)
Probable PKDL (N = 76)	Microscopy	39.47% (28.44%-51.35%) (30/76)	N/A
	Q-qPCR	68.42% (56.75%-78.61%) (52/76)	100% (95.26%-100%) (76/76)
	Q-LAMP	72.37% (60.91%-82.01%) (55/76)	100% (95.26%-100%) (76/76)
	B&S-LAMP	57.89% (42.02%-69.14%) (44/76)	100% (95.26%-100%) (76/76)

Among all PKDL individuals (n = 76), 65 (85.5%) cases were found to be positive by either of microscopy, qPCR, or LAMP, whereas 11 cases were negative by these methods. Of the 65 positive cases, 61 (81.3%) were detected by molecular methods whereas only 4 (6.2%) were positive by microscopy alone. Of the three diagnostic methods evaluated, microscopy had the lowest sensitivity of 39.5% (95% CI: 28.44–51.35%) (Table 2). Although 61 cases were detected by the composite of all molecular methods, qPCR detected only 52 cases and had a sensitivity of 68.4% (95% CI: 56.75–78.61%) (Fig. 3). As expected, Q-LAMP had higher sensitivity (72.37%) and detected more PKDL cases than the B&S-LAMP assay. With this elevated sensitivity, the Q-LAMP detected an additional 11 cases over the 44 cases detected by B&S-LAMP assay.

Excellent agreement was observed between Q-LAMP and Q-qPCR (k = 0.905 (Table 3), while B&S-LAMP showed moderate agreement with Q-qPCR (k = 0.60). Agreement declined when the comparative method was Q-LAMP (k = 0.349). Of note, a significant discordance (P = 0.04) was observed between the two DNA extraction methods used for the LAMP assay. As all of the endemic healthy controls (EHCs) were negative, thus an absolute (100%) specificity was observed for each of these molecular methods.

**Table 3:** Agreement between the assays while DNA isolated through a commercial kit and an in-house boil & spin method.

	Kappa(k)	Agreement	McNemar (P value)
Q-LAMP Vs B&S-LAMP	0.349	Weak	0.04
Q-qPCR Vs B&S-LAMP	0.600	Moderate	0.09
Q-LAMP Vs Q-qPCR	0.905	Excellent	0.25

### Correlation between parasite load and diagnostic performance

**Figure 4:**
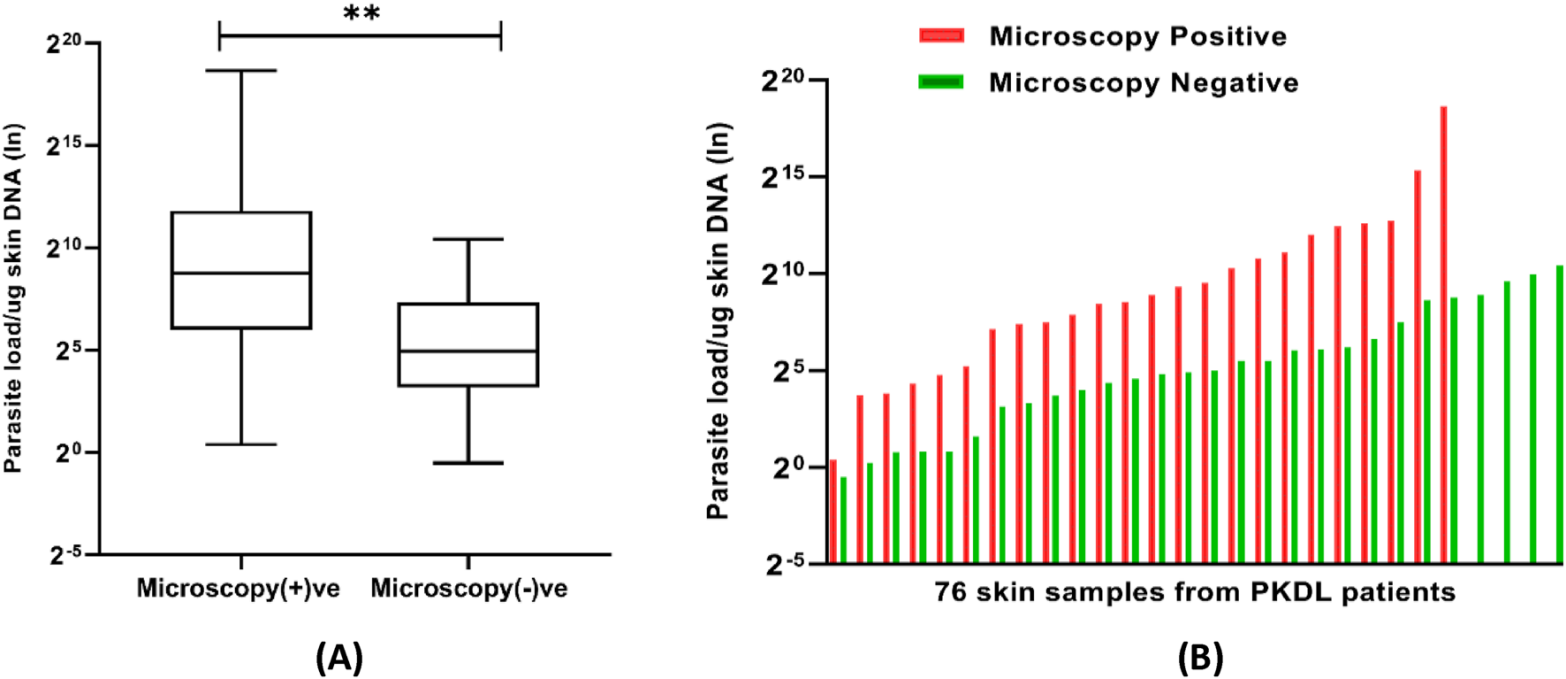
(**A**) Difference in parasite loads between microscopy positive and negative cases. (**B**) Distribution of parasite loads among microscopy positive and negative cases.

**Figure 5:**
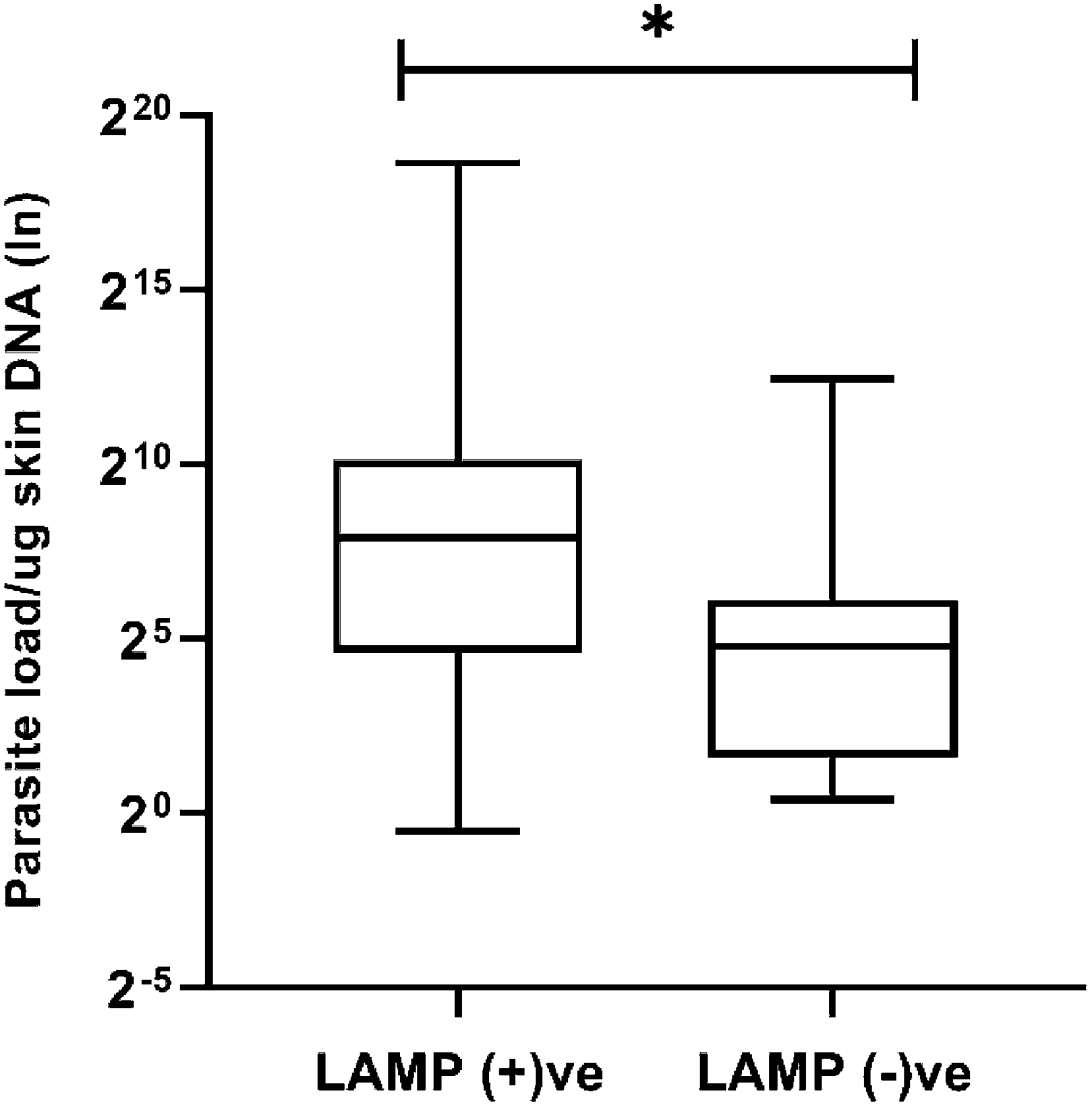
Difference in parasite loads between boil &spin LAMP positive and negative cases.

**Figure 6:**
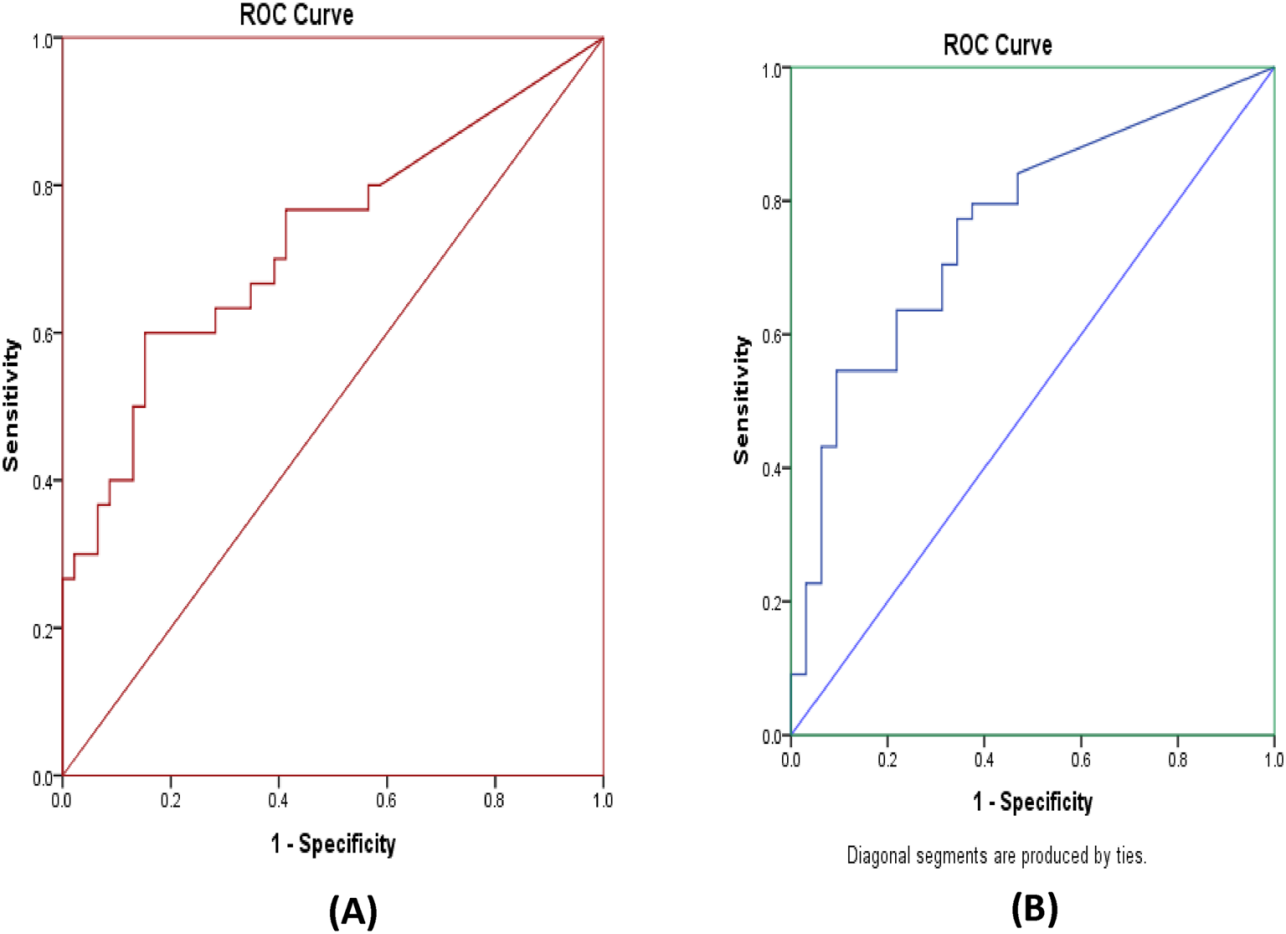
Receiver operating curve analysis to determine the trade-off parasite load of PKDL cases for being diagnosed as positive in Microscopy (**A**—left) and B&S-LAMP Assay (**B**—right).

As expected, the parasite load determined through qPCR significantly influenced the outcome of microscopy and LAMP assay, with a significant difference (p < 0.001) observed in parasite loads among samples from patients that had a positive microscopy result versus those that had a negative result (Fig. 4A). The mean parasite loads for microscopy positive and negative cases were 20,378 and 187 parasites/µg tissue DNA, respectively. Further analysis identified that an elevated level of parasites was observed for all microscopy positive cases, whereas only four skin samples with higher parasite loads were assessed as negative by microscopy (Fig. 4B). Similarly, we observed a significant difference (p < 0.05) in parasite loads between LAMP positive and negative cases when the boil and spin DNA extraction method was followed (Fig. 5). Most of the B&S-LAMP positive cases had higher parasite burden than their negative counterparts, whereas only four individuals negative by qPCR were found to be positive in B&S-LAMP. Notably, all individuals positive by qPCR were positive for Q-LAMP, an observation that we attribute to the positive correlation between the parasite load and the performance of the LAMP assay. The ROC analysis found the cut off parasite loads for B&S-LAMP and microscopy as 3 and 6 parasites/µg tissue DNA, respectively, where the parasite loads quantified by qPCR in skin samples ranged from 7 × 10^–1^ to 4 × 10^5^ parasites/µg tissue DNA (Fig. 6).

### Bayesian latent class modeling

In Bayesian latent class modeling, the estimated sensitivities for Q-LAMP (90%, 95% CI: 80–98%) and qPCR (90%, 95% CI: 84–95%) were found to be very similar (Table 4). As expected, microscopy had poor sensitivity (50%, 95% CI: 42–58%), whereas B&S-LAMP showed a moderate sensitivity (67%, 95% CI: 54–79%). Both qPCR and microscopy had 100% specificity, with no positive results when EHCs were assessed (Table 4). Lower specificity was observed for Q-LAMP (89%, 95% CI: 67–99%) and B&S-LAMP (74%, 95% CI: 50–91%). Finally, the estimated prevalence of confirmed PKDL patients was 77% (95% CI: 66–87%) among the total 76 PKDL cases.

**Table 4:** Estimated sensitivity and specificity of the diagnostic tests for LD bodies in 76 probable PKDL patients, under a Bayesian latent class modeling approach.

Diagnostic methods	Sensitivity (95% CI)	Specificity (95% CI)
Q-LAMP	90% (80–98%)	89% (67–99%)
B&S-LAMP	67% (54–79%)	74% (50–91%)
Q-qPCR	90% (84–95%)	100% (100–100%)
Microscopy	50% (42–58%)	100% (99–100%)

## Discussion

In the last decade, the kala-azar elimination program has taken major strides towards elimination of VL from endemic areas. However, with a persistent ability to transmit parasites PKDL patients present a continued challenge to the elimination campaign. Although the infectiousness of PKDL cases towards sand flies has long been debated, transmission from PKDL patients was recently proven through the xenodiagnosis studies^10,26^. Early diagnosis and treatment of both VL and PKDL are therefore needed to break the *L. donovani* transmission cycle^12,27^. Medley et al.^28^ estimated that approximately 25% of total VL cases stem from PKDL cases, highlighting the need for prompt identification and treatment of PKDL to achieve the VL elimination goal. While qPCR has become a mainstay for diagnosis and prognosis of VL, experts have recently underscored the need for an alternative ultra-sensitive molecular tool for diagnosis of PKDL^12,29^. Isothermal techniques including LAMP, recombinase polymerase amplification (RPA) assay, and nucleic acid sequence based amplification (NASBA) have made detection of parasite DNA simple, rapid, and cost-effective^30^. The LAMP assay developed by Eiken is highly sensitive for diagnosing VL and CL^21,22,31^. However, there are no data to determine the efficacy of this particular LAMP assay for the diagnosis of PKDL. Considering the different endemic settings, available healthcare facilities, and current guidelines, we determined the diagnostic value of the investigational assay in individuals with PKDL.

LAMP performed better than qPCR when QIAGEN was used for DNA extraction. This could be attributed to the dual target amplicons in LAMP which include minicircle kinetoplast DNA (kDNA) and 18S ribosomal DNA (rDNA) of the parasite genome, compared to the qPCR assay which targets the REPL repeats of 18S rRNA genes of the parasite genome. Additionally, the LAMP assay has a higher analytical sensitivity (LOD = 0.001 parasite/µl) than qPCR^19,21^. With similar results, two studies have reported the efficiency of a kDNA-dependent LAMP assay on the ISC^32^. A limitation of both studies, however, was that the majority of PKDL patients evaluated had the less common papular and nodular rashes that present with a high parasite burden, compared to the majority that present with macular rashes and few parasites in the skin such as those evaluated in the present study^33,34^.

DNA isolation methods have a direct impact on the efficiency of downstream molecular diagnostic methods^35,36^. In our evaluations we observed a higher efficiency of LAMP assay when the LAMP reaction was performed using DNA isolated with a commercial kit (Qiagen) compared to the in-house boil-spin method. Since the boil-spin method isolates crude DNA from the clinical sample, the presence of the impurities (e.g., tissue debris, hemoglobulin, protein etc.) in DNA can hinder target amplification. In contrast, the commercial spin-column method isolates purified DNA as the process involves use of protease K and buffer solutions for multiple washing steps which favors optimal amplification of the target amplicon. Indeed, consistent with our findings, an earlier study by Chowdhury et al.^35^ showed that the boil and spin method is less efficacious than a commercial spin-column method in detecting LD DNA by Recombinase Polymerase Amplification assay. Moreover, the skin samples used for DNA extraction by different methods were collected from different lesion sites and thereby represents a potential contributing factor in the disagreement between B&S-LAMP and Q-LAMP. Likewise, in our previous study we observed discrepancies in qPCR results while skin samples were collected from variable lesion sites^14^.

In this study the sensitivity of qPCR was lower than previous studies, a result that might be attributed to the use of a different skin sampling method. In contrast to the present study, our previous studies collected skin biopsies that are likely to contain more parasites as a larger sample is obtained that also contains the sub-cutaneous layer^14,19^. Thus, skin biopsies likely also collect numerous tissue resident immune cells (e.g., neutrophils, APC, keratinocytes, Langerhans cells, macrophages etc.) harboring the parasites. In contrast, here a snip skin was collected using a scalpel such that sampling contains mostly the epidermis.

Similarly, a nominal decline in the sensitivity of microscopy was observed relative to our previous study^14^. As indicated in earlier studies, the sensitivity of microscopy can differ markedly (4–71%) depending on the type of lesions and the microscopic methods used to detect the LD bodies^6,13^. Again, however, this small difference in the sensitivities between these studies could be attributed to the alternative sampling procedure. It is also worth noting that most of the microscopy positive cases were found to be grade 1 (n/N:24/30), indicating the presence of few parasites beneath the skin. Moreover, a significant difference in parasite load was observed between microscopy positive and negative samples through qPCR (Fig. 4). The study findings infer that the degree of chronicity of the infection is correlated to the performance of the microscopy.

Although the findings of the current study are consistent with the encouraging diagnostic performance of the various investigative methods reported in the previous studies^14,22,31^, To address the absence of a true gold standard we used a Bayesian latent class modeling approach to estimate sensitivity and specificity of the diagnostic methods, accounting for the imperfect accuracy of reference diagnostics. Under this approach, the estimated sensitivity for each of the method was found parallel to the conventional approach. These findings were consistent regardless of model specification although specificity was more modest for the investigational methods in the latent class analysis.

In addition to determining the performance of different diagnostic tools for PKDL, our study design enabled us to further explore the influence of different VL treatment modalities towards progression to PKDL. The median timeline for development of PKDL following each of the VL treatment modalities was found to be congruent with the findings of our previous studies^9,14^. However, no associations were observed between the number of rashes and parasite load with different treatment regimens in developing PKDL. Similar to our previous study, no correlation was found between the parasite load and time between VL treatment and onset of PKDL (P = 0.27)^14^. Further, differences among the various treatment modalities in terms of parasite load during diagnosis of the PKDL cases were not significant (p > 0.05). Interestingly, a positive association was observed between the score in manikin and parasite load or degree of chronicity. Recently Moulik et al.^37^ showed the advantages of qPCR in treatment monitoring of PKDL patients which further invokes the application of the investigative assays for similar purpose.

In our study we showed that the performance of each method likely depends on the chronicity of the infection or parasite load. Without exception, each method showed a significant association between parasite load and positivity and thus our findings substantiate that parasite load significantly influences the efficiency of the diagnostic methods (Figs. 4, 5). In addition to these biological factors, the interval between the appearance of patent signs for PKDL and diagnosis of the patients at healthcare centers might be critical for efficiency of the diagnostic methods. Recently several vertical programs for kala-azar elimination have demonstrated a synergy and have been successful in improving the health seeking behavior of the VL/PKDL patients by persuading the patients to seek treatment earlier and while they have a less chronic condition^38^.

In this study, a total of 76 PKDL cases were enrolled who responded well following their treatment with Miltefosine. During enrolment, all of these enrollees were identified as putative PKDL cases carrying *leishmania* infection which necessitated their treatment as per the national guidelines. Our recent xeno-diagnosis study established the transmission potential of qPCR/microscopy confirmed PKDL cases which invokes further questions regarding the transmission capability of microscopy or qPCR negative PKDL cases^10,26^. The above evidence warrants meticulous decision for treatment of PKDL cases through using current laboratory based diagnostic algorithms. However, putative PKDL cases that remain unclear through clinical along with serological algorithm require confirmation through further laboratory-based methods and, in such instances, molecular methods have been found to be more accurate for this purpose^39–41^. Moreover, recent literature reviews have emphasized that the molecular methods can be used as a treatment monitoring tool for PKDL^42,43^. Such molecular methods may eventually become a prerequisite to control enigmatic cases through proper diagnosis and appropriate treatment.

A major limitation of our study is the use of multiple skin samples for different diagnostic methods. Therefore, the possibility of inter and intra-method variability in diagnostic performance cannot be excluded. However, homogeneous DNA samples were used to perform LAMP and qPCR and this ensured the absolute comparison between the molecular methods. Moreover, we collected the skin snip from prominent lesional sites to minimize the heterogenicity among the samples. In contrast to the effect of heterogeneous sampling, inclusion of the pool of macular cases might have underestimated or compromised the sensitivity of the diagnostic methods. Earlier studies explicitly showed greater performance of diagnostic methods for papular and nodular PKDL in comparison to macular cases^13,14,34,35^. In addition, blood samples were used to determine the specificity of the diagnostic methods, as the ethical reason/constraint circumvented the collection of skin samples from healthy controls which is aligned with our previous study^14,35^.

The promising efficacy of LAMP assay found here substantiates its application as an alternative molecular assay of PCR/qPCR for diagnosing the PKDL cases, especially the macular presentation. Moreover, our findings provide a critical insight on proper sampling and DNA isolation method for optimum efficiency of this design-locked LAMP assay with minute chance of carry-over contamination. Despite being cheap and rapid, the in-house boil-spin method requires further methodological improvisation to facilitate the diagnosis of PKDL at point-of-need through this ultra-sensitive isothermal assay. Recently, we showed that RPA assay, an isothermal molecular technique is field deployable if the method is incorporated in mobile suitcase laboratory^44^. Since LAMP is simple, cheap, design-locked and easy to perform, therefore, such integration might bring this isothermal technique a step ahead towards point-of-care application.

## Methods and materials

### Study sites and participants

The study was conducted at the Surja Kanta Kala-azar Research Centre (SKKRC), Mymensingh Hospital, Bangladesh and The Emerging Infections and Parasitology Laboratory at the International Centre for Diarrheal Disease Research (icddr,b), Dhaka, Bangladesh. All participants were enrolled between June 2016 and April 2017 and were resident in the highly endemic district of Mymensingh. Individuals with presumptive PKDL with or without history of VL, living in endemic areas, and presenting with a macular, papular, or nodular skin manifestation without loss of sensation, were clinically evaluated at SKKRC. After screening, individuals were tested for anti-*Leishmania* antibodies using an immunochromatographic RDT (Kalazar Detect™ Rapid Test for Visceral Leishmaniasis, InBios International, Inc., USA, hereinafter rK39 RDT) as per the national guidelines for VL and PKDL diagnosis^45^. Individuals returning a positive rK39 RDT were invited to participate in the study and written informed consent was obtained. Seventy-six participants with or without a history of VL, presenting with symptoms of PKDL, and a positive rK39 RDT were enrolled in the study as probable PKDL patients.

Skin rashes of participants with probable PKDL were scored by the study physician according to the method described by Mondal et al.^46^, and three skin-snip samples were collected through scalpel from each participant as per the standard operating procedure such that sampling contains mostly the epidermis. Moreover, the prominent leisonal sites were selected from skin snips collection which are more likely to harbor leishmania parasites such that the chance of heterogenicity among the samples decreases. Among three skin-snip samples collected from each participant with probable PKDL, one skin-snip sample was used for skin smear and two skin-snip samples were preserved in NET 10 buffer (50 mM NaCl, 125 mM EDTA, 50 mM Tris–HCl [pH 7.6]) for subsequent DNA extraction. Samples were stored at − 80 °C until transfer to icddr,b in cold chain. Both microscopy and qPCR were performed for each of the probable PKDL cases. Probable PKDL cases positive by microscopy or qPCR were considered as confirmed PKDL cases. Following enrollment, all of the PKDL patients were referred to the SKKRC for treatment according to national guidelines^45^.

Age and sex matched clinically healthy household contacts (n = 76) without history of VL or PKDL and negative by DAT (< 1600) and rK39 RDT were enrolled as endemic healthy controls (EHCs). Venous blood (2 ml) was collected from 76 EHCs for DAT and DNA extraction.

### rK39 rapid diagnostic test (RDT)

The Kala-azar Detect™ Rapid Test kit (Inbios International, Inc., USA) was performed on serum samples from participants with suspected PKDL according to manufacturer’s instructions. Briefly, 20 µL serum was added to the sample pad and 2–3 drops (150 µL) of chase buffer were added. The result was read after 10–20 min. Test strips showing both control and test lines were considered positive. Test strips showing only the control line were considered negative. If the control line was absent, the test was considered invalid and repeated^47^.

### Direct agglutination test (DAT)

The DAT was carried out at SKKRC as previously described^48^. Following a dilution of sera 1 in 200, the samples were further diluted in eight two-fold serial dilutions. Where samples did not react in the first dilution, the end titer was read as < 1:200. Where samples still reacted at the final dilution, the end titer was read as > 1:25,600. The threshold for a positive DAT was ≥ 1:1600.

### Microscopy

Giemsa-stained smear was prepared from skin-snip biopsies and examined under a light microscope (1000X magnification). Two expert microscopists examined the slides to identify the Leishman-Donovan (LD) bodies. Discrepancies were resolved by an independent microscopist. LD bodies were graded in accordance with the World Health Organization grading system^49^. Slides were scored as grade 0 (0 parasites in 1000 fields), grade 1 (1–10 parasites in 1000 fields), grade 2 (1–10 parasites in 100 fields), grade 3 (1–10 parasites in 10 fields), grade 4 (1–10 parasites per field), grade 5 (10–100 parasites per field), or grade 6 (> 100 parasites per field)^14^.

### DNA extraction

#### Silica spin column-based method (QIAGEN)

DNA was extracted using DNeasy Blood & Tissue Kits (QIAGEN, Hilden, Germany) according to manufacturer’s instructions, with a minor modification: skin-snip materials were incubated at 37 °C overnight after addition of lysis buffer (ATL) and proteinase K. The following day, the material was homogenized and incubated at 56 °C for two hours before purification according to the manufacturer’s instructions. DNA was eluted in 200 µL PCR grade water or elution buffer. Extracted DNA samples were stored at − 80 °C or − 20 °C.

#### In-house Boil & Spin method (B&S)

Lysis buffer (400 mM NaCl, 40 mM Tris pH 6.5, 0.4% SDS)^50^ and proteinase K (QIAGEN, Hilden, Germany) was added with skin-snip samples and were incubated at 37 °C overnight. The following day, skin samples were homogenized then incubated at 70 °C for 15 min. After incubation, the mixture was vortexed, spun and incubated for 5 min at 95 °C before centrifugation for 3 min at 10,000 g. After centrifugation, 30 μL of supernatant was transferred to a dilution tube containing 345 μL of PCR grade water and stored at − 80 °C or − 20 °C.

### qPCR

A qPCR assay was performed according to a method described by Vallur et al.^51^, using TaqMan® primers and probes targeting *Leishmania infantum* DNA repeat region (REPL-repeats; GenBank Accession Number L42486.1) specific for *L. donovani* and *L. infantum* and synthesized by Applied Biosystems. Twenty microlitres reaction mix was prepared containing 5 μL template, 10 μL of TaqMan® Gene Expression Master Mix (2×), 1 μL primer–probe mix and PCR grade water. Amplification was performed on a Bio-Rad CFX96 iCycler system with the following reaction conditions: 10 min at 95 °C, followed by 45 cycles of 15 s at 95 °C and 1 min at 60 °C. To quantify the parasite load of each sample, each run included one standard curve with ten-fold DNA dilutions ranging from the equivalent of 10,000 to 0.1 parasites per reaction. Each run also included one reaction with molecular grade water as a negative control. Each DNA sample was analyzed in duplicate and in case of an indeterminate result, one additional analysis was performed. Samples with cycle threshold (Ct) > 40 were considered negative.

### Loop-mediated isothermal amplification (LAMP)

Loopamp™ *Leishmania* Detection Kit (Eiken Chemical Co. Ltd., Japan) targeting two regions of the *Leishmania* genome (18SrRNA gene and the kinetoplast DNA minicircles) was conducted according to manufacturer’s instructions. LAMP buffer (27 μL) with DNA (3 µL) extracted by QIAGEN, or 30 μL template DNA extracted by B&S were added to each LAMP tube, supplied as string of 8 tubes with caps containing lyophilized master mix. A positive control and 30 μL LAMP buffer as negative control was included in every run. After addition of the template, tubes were closed, the string was turned upside down and inverted twice. The string was placed cap-side down on the bench for 2 min for reconstitution of the dried master mix. Following reconstitution, the solution was centrifuged, and the string was incubated in the Loopamp™ LF-160 incubator (Eiken Chemical Co. Ltd., Japan) at 65 °C for 40 min, then 80 °C for 5 min. After incubation, the string was placed in a visualization unit integral to the incubator and the results were visualized under blue LED light. The assay was considered positive when fluorescence was observed by two independent technicians. Discordance in interpretation was resolved by a third interpreter.

### Statistical analysis

Standard statistical methods including contingency tests and Binomial confidence intervals were followed to determine the sensitivity and specificity of the LAMP with 95% CI. In this study, the diagnostic efficacy of LAMP assay was determined against both confirmed PKDL and probable PKDL. Parametric and non-parametric tests were performed based on the distribution of data. Cohen’s kappa coefficient (k) and McNemar’s test were performed to determine the concordance and discordance between diagnostic methods. Categorical variables were presented as absolute number and percentage. Continuous variables were summarized using means with standard deviations and non-continuous variables were presented by median with interquartile range (IQR). Receiver operating characteristic (ROC) curve analysis was performed to determine the minimum parasite load in microscopy positives and LAMP assay. Kaplan–Meier survival analysis was performed to estimate the median time to diagnosis of PKDL following the treatment of VL. All statistical analyses were performed using SPSS (Version 25.0) and GraphPad Prism (Version 8.1.2). P value < 0.05 was considered as statistically significant.

### Latent class analysis (LCA)

Sensitivity and specificity of each test was estimated using a Bayesian latent class modeling framework^52^. We incorporated prior estimates for accuracy of PCR and microscopy from published literature^14^ and used noninformative beta priors for the LAMP assays and disease prevalence. The observed data were assumed to follow a multinomial distribution with probabilities determined by the disease prevalence, sensitivity, and specificity for each combination of diagnostics. We used Markov Chain Monte Carlo sampling with 100,000 iterations, discarding the first 50,000, and reporting the median and 95% credible interval of the posterior distributions. Modeling was performed using RStan.

### Ethics

The study was approved by icddr,b Institutional Review Board (IRB), protocol number PR-14093. Written informed consent form was obtained from each of the participants, or from the parents/guardian in the case of minors, prior to enrollment in the study. All research pertaining to the study was performed in accordance to the Declaration of Helsinki.

## Data Availability

The data used to support the results of this study can be obtained from the corresponding author upon reasonable request.
